# Enhancing anti-viral neutralization response to immunization with HIV-1 envelope glycoprotein immunogens

**DOI:** 10.1038/s41541-023-00774-z

**Published:** 2023-11-24

**Authors:** Shamim Ahmed, Durgadevi Parthasarathy, Rachael Newhall, Tashina Picard, Morgainne Aback, Sneha Ratnapriya, William Arndt, Widaliz Vega-Rodriguez, Natalie M. Kirk, Yuying Liang, Alon Herschhorn

**Affiliations:** 1 Division of Infectious Diseases and International Medicine, Department of Medicine, University of Minnesota, Minneapolis, MN 55455 USA; 2 School of Dentistry, University of Minnesota, Minneapolis, MN 55455 USA; 3 Department of Veterinary and Biomedical Sciences, College of Veterinary Medicine, University of Minnesota, St Paul, MN 55108 USA; 4 Institute for Molecular Virology, University of Minnesota, Minneapolis, MN 55455 USA; 5 Microbiology, Immunology, and Cancer Biology Graduate Program, University of Minnesota, Minneapolis, MN 55455 USA; 6 The College of Veterinary Medicine Graduate Program, University of Minnesota, Minneapolis, MN 55455 USA; 7 Molecular Pharmacology and Therapeutics Graduate Program, University of Minnesota, Minneapolis, MN 55455 USA

**Keywords:** Vaccines, Virology

## Abstract

An effective human immunodeficiency virus type I (HIV-1) vaccine that robustly elicits broadly neutralizing antibodies (bnAbs) against HIV-1 envelope glycoproteins (Envs) to block viral entry is still not available. Thus, identifying triggers for elicitation of different types of anti-HIV-1 Env antibodies by vaccination could provide further guidance for immunogen design and vaccine development. Here, we studied the immune response to HIV-1 Env immunogens in rabbits. We show that sequential immunizations with conformation-specific Env immunogens can elicit low titer but broad neutralization responses against heterologous, neutralization-resistant (tier 2/3) transmitted/founder (T/F) HIV-1 strains. More importantly, an mRNA vaccine candidate that could mediate the presentation of a cytoplasmic tail-deleted (ΔCT) HIV-1_AD8_ Env immunogen on virus-like particles significantly increased the neutralization response. This strategy shifted the type of elicited antibodies, decreasing the level of binding to soluble Envs while significantly increasing their overall viral neutralization activity. The breadth and potency of neutralizing response against heterologous, T/F HIV-1 strains significantly increased in a subset of rabbits. Efficient neutralization activity was associated with high cellular immune responses specific to HIV-1 Envs. These results help to understand the immune response to different immunization schemes and will allow developing new approaches to selectively manipulate the type of humoral immune response by specific vaccination.

## Introduction

Human immunodeficiency virus type 1 (HIV-1) continues to spread with ~37 million people living with HIV-1 worldwide in 2021 (www.who.org). Despite an abundance of approaches and available modalities with proven efficacy to prevent HIV-1 infection, approximately 1.5 million individuals were infected with HIV-1 in 2020. High numbers of newly infected individuals have led to intensive ongoing and innovative efforts for developing effective ways to prevent HIV-1 infection. A protective HIV-1 vaccine is yet not available but multiple approaches that emerged in parallel provide new insights to guide vaccine development. A recent study proved the feasibility/proof of concept of germline-targeting vaccine priming approach^1^. In parallel, results from the first-in-human antibody-mediated prevention trial HVTN 703/HPTN 081 provide an estimate for the potency of elicited antibodies that would be required to prevent HIV-1 acquisition^2,3^. Additionally, correlates of protection in the RV144 trial, which showed modest and temporal HIV-1 protection, suggest that non-neutralizing antibodies target HIV-1 gp120 V1/V2 loop may contribute to protection from HIV-1 infection^4^.

Numerous studies in non-human primates and vaccine efficacy trials in humans correlated antibodies against the HIV-1 envelope glycoproteins (Envs) and HIV-1 protection^4–7^. HIV-1 Envs mediate viral entry into target cells and are the sole target of neutralizing antibodies. HIV-1 Envs are assembled into a trimeric spike that is composed of three gp120 exterior subunits associated noncovalently with three gp41 transmembrane subunits^8^. Each Env subunit mediates specific activity: the surface gp120 subunit binds the host CD4 receptor and CCR5/CXCR4 coreceptors, and the transmembrane gp41 facilitates membrane fusion. Either spontaneously or in response to receptor binding, HIV-1 Envs can transit from a metastable, high-potential energy closed conformation (State 1) to downstream states (States 2 and State 3) that are associated with multiple structural rearrangements^9,10^. Recent identification and isolation of broadly neutralizing antibodies (bnAbs) against HIV-1 Envs pinpoint target sites of Env vulnerability. Although direct as well as potential indirect mechanisms of HIV-1 escape from some of the bnAbs in vivo have been recently reported^11–13^, bnAbs still provide promising guidance to the type and properties of elicited antibodies that may provide protection. Anti-HIV-1 bnAbs can be classified according to their preference to recognize specific Env conformations^9,14–16^. Most bnAbs that target the CD4 binding site (CD4bs) as well as those targeting the V1/V2 loop of gp120 prefer to neutralize the Env closed conformation (State 1). These bnAbs still neutralize lab-adapted HIV-1 Envs, which are considered fully open. However, these bnAbs typically lose anti-viral activity against primary HIV-1 Envs of all major clades that are stabilized in downstream conformations by genetic modifications of residues that naturally restrain HIV-1 Env closed conformation^9,15,16^. In contrast, bnAbs targeting the membrane-proximal external region (MPER) of gp41 preferentially recognize downstream Env conformations (State 2 and State 3), probably because their epitope, which is located near the viral membrane, is more accessible on the open Env conformations^14,17^. In addition to these two groups, some bnAbs can recognize and neutralize multiple Env conformations^18^.

## Results

As some bnAbs selectively neutralize specific HIV-1 conformations, we hypothesized that presentation of multiple Env conformations to the immune system may be beneficial for elicitation of diverse antibodies that preferentially recognize different conformations as well as for elicitation of broad antibodies that recognize multiple Env conformations. Such presentation could potentially address heterogeneity of Env closed conformation. Many HIV-1 Envs of primary strains, including transmitted/founder (T/F) HIV-1 strains that can establish HIV-1 infection in vivo, prefer to be tightly closed but some of these Envs are incompletely closed and they slightly or transiently expose internal epitopes^19^. In a preliminary study, we immunized 2 rabbits with a sequential combination of 3 soluble SOSIP Env trimers (Supplementary Fig. 1). We expressed and purified the 1059, BG505, and B41 Envs as soluble SOSIP trimers and confirmed different conformational states of 1059 and BG505 SOSIPs as detected by the differential exposure of 17b epitope, which overlaps with the coreceptor binding site in gp120 (Supplementary Fig. 2). The immunization scheme included priming with 1059-SOSIP (incompletely closed Env; Ref. ^19^), which are based on HIV-1 Envs that are currently being tested in a human DNA-based vaccine trial (HVTN 106 trial; NCT02296541), followed by boosting with a combination of BG505/B41 SOSIPs (tightly closed Envs) and sequential additional boosting with a combination of the 3 Env immunogens. We reasoned that after immunization with each type of SOSIP separately (1059-SOSIP, which is incompletely closed and BG505 SOSIP, which is tightly closed), immunization with both may facilitate the induction of a broader antibody response. We collected blood at intervals of 2–7 weeks and analyzed the antibody response in the serum. We detected a strong antibody binding response to 1059-SOSIP in the serum of both rabbits after 2 weeks and this response was sustained at least until week 31. Antibodies developed over time neutralized viruses pseudotyped with the easy-to-neutralize HIV-1_SF162_ Envs (tier 1) and the efficiency of neutralization was gradually increased with the additional immunizations (Supplementary Fig. 1). We next tested the ability of rabbit sera to neutralize viral entry mediated by 13 available T/F HIV-1 Envs, which have been previously isolated and published^20^. T/F strains can establish HIV-1 infection in vivo, express about 1.9-fold higher Envs on their surface, typically resist interferon-induced protection, and are the main target of HIV-1 vaccines for prevention^20,21^. We detected low titer but broad inhibition of T/F entry in the serum of rabbit 1. A low titer (1/20) of rabbit 1 serum at week 25 neutralized >50% of pseudoviral entry of 9 out of the 13 T/F strains and this number was decreased to 6 out of 13 T/F strains for serum from blood collected at week 31. In contrast, the serum of rabbit 2 showed only marginal effects on the entry of some of these T/F strains (Supplementary Fig. 3).

**Fig. 1:**
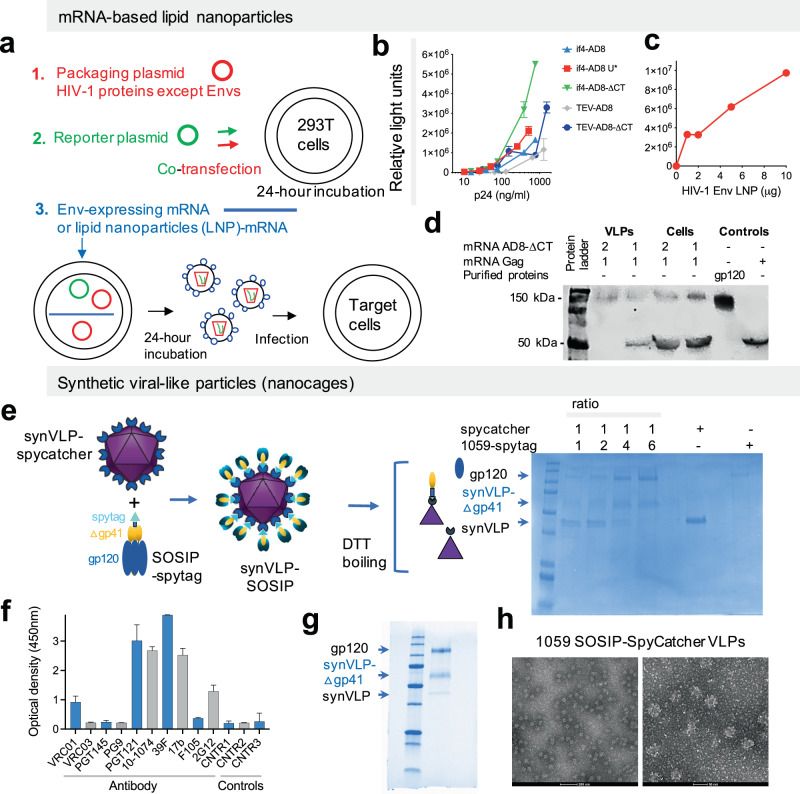
Preparation and validation of mRNA-based lipid nanoparticles (mRNA-LNPs) and synthetic virus-like particles (syn-VLPs) for delivery of HIV-1 vaccine candidates. **a** A scheme for preparation of pseudoviruses (PVs) using purified mRNA (Supplementary Fig. 4) or mRNA-LNP for expression of HIV-1 Envs. **b** Infection of Cf2Th-CD4^+^/CCR5^+^ target cells by PVs prepared with in vitro transcribed and purified mRNA for HIV-1 Env expression. U*, pseudouridine; IF4, an aptamer that binds eukaryotic translation initiation factor 4 G; TEV, sequence derived from the 5’ UTR of the tobacco etch virus. **c** Similar to (**b**) but we used mRNA-LNP (AD8-ΔCT) to prepare PVs instead of purified mRNA and the PV infectivity was plotted against increasing amounts of mRNA-LNP used. **d** mRNA-mediated co-expression of HIV-1_AD8_ ΔCT Envs and HIV-1 Gag in 293 T cells produces virus-like particles. Western blot of concentrated supernatant-containing VLPs and of mRNA-transfected cells using serum from an HIV-1 infected Individual and secondary, HRP-conjugated anti-human IgGs (1:10,000 dilution; cat# 709-036-098; Jackson ImmunoResearch Inc.). Numbers indicate mRNA ratio. **e** Optimization of syn-VLPs preparation. Different ratios of 1059-SOSIP-Spytag and SpyCatcherVLP were mixed and incubated overnight following by SDS-PAGE analysis to determine optimal conjugation ratio. The synVLP-spycatcher shape was taken from Brune KD et al. Sci. Rep. 2016; 6:19234 (distributed under the terms of Creative Commons CC BY license). **f** ELISA of binding of 0.625 µg/ml of different antibodies to 1059-spytag trimer (unconjugated). CNTR1 and CNTR2, VRC01 and 2G12 binding to unrelated proteins (Spycatcher-VLP), respectively; CNTR3, secondary antibodies only. **g** SDS-PAGE of 1059-SOSIP-Spytag and SpyCatcherVLP conjugated at 6:1 ratio and purified by size-exclusion chromatography. **h** Electron microscopy images of assembled 1059-SOSIP-SpyCatcher VLPs that were negatively stained (see also Supplementary Fig. 6). All gels and blots were derived from the same experiment and were processed in parallel. Results are from single (**b, f**) or representative of 2 (**c**) experiments performed in duplicate (mean ± range).

To test our immunization approach on a larger number of rabbits, we first modified 2 platforms to deliver HIV-1 Env immunogens: mRNA-LNPs^22,23^ and synthetic icosahedral nanocages^24–26^ (synthetically engineered viral-like particles; synVLP). Both delivery methods have been developed and recently published by others for delivery of different immunogens^22–26^ and we applied published data and knowledge to build or modify the related vectors to deliver HIV-1 Env immunogens. We built and optimized new vectors for in vitro transcription of mRNA for the expression of HIV-1_AD8_ Envs (Supplementary Figs. 4 and 5), derived from an HIV-1 strain (AD8) that has been extensively studied in a non-human primate model of HIV-1 infection^27^. We confirmed the level of Env expression in 293 T cells and validated entry-compatibility of mRNA-mediated Env expression by producing PVs using the mRNA as a source for the Envs (Fig. 1). HIV-1_AD8_ PVs were highly infectious and, notably, removal of the cytoplasmic tail (ΔCT) of HIV-1_AD8_ Envs significantly increased viral infectivity, indicating that the HIV-1_AD8_ ΔCT Envs were maintaining entry-compatible conformation on the pseudovirus surface. mRNA-LNPs that mediate expression of HIV-1_AD8_ ΔCT Envs successfully supported the production of infectious PVs with dose-dependent infectivity within a range of 1 to 10 μg of mRNA-LNP (Fig. 1). In parallel, we built a multi-valent display of 1059-SOSIP on synthetic nanocages using an available published system that is based on the Spytag-SpyCatcher technology (Fig. 1)^25,26^.

**Fig. 2:**
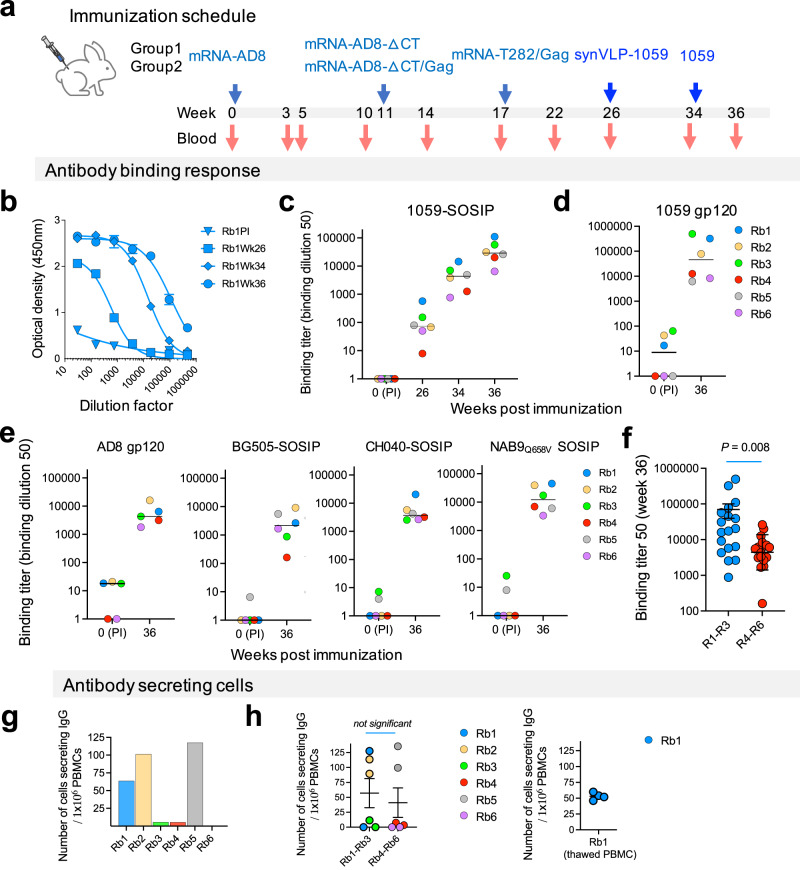
Elicitation of high-titer binding antibodies to conformation-specific HIV-1 Env immunogens delivered by mRNA-LNP and syn-VLPs. **a** immunization scheme of 6 rabbits with conformation-specific immunogens. T282 Env was previously reported to induce broad antibody response after transmission to humans (Kouyos et al. Nature 561, 406–410, 2018) and therefore it was included in our study. **b** Binding of IgG-containing sera from rabbit 1 (Rb1) to soluble HIV-1_1059_ SOSIP Env. Sera were collected at specified time points, and IgG binding was measured by ELISA. **c**–**e** Binding titer of sera from all 6 rabbits to 1059-SOSIP (**c**), 1059 gp120 (**d**), and different homologous and heterologous Envs (**e**) at indicated time points after immunization. NAB9 SOSIP was not well expressed, and we used the NAB9_Q658V_ mutant that contains a trimeric stabilization mutation (Rawi, R. et al. *Cell Reports 33, 108432, 2020)* was used. **f** Statistical analysis of the differences between the binding titer of group 1 and group 2 rabbits to different HIV-1 Envs. **g** Average numbers of antibody-secreting cells (ASC) in the blood of each rabbit. **h** Left - comparison of the number of ASC in the 2 groups of rabbits. Right - due to high variation in the results of cells from Rb1, the experiment was repeated with frozen/thawed PBMCs. Results of each individual sample with median (**c**–**e**) or mean ± sem (**f**, **h**) of all samples are shown. *P*, two-tailed *P* value of Mann–Whitney *U*-test. PI, pre-immune.

We next immunized 6 rabbits with a combination of different vectors that delivered HIV-1 Env immunogens at different conformations. All rabbits were primed with mRNA-LNPs, boosted twice with different mRNA-LNPs, and then sequentially boosted with synVLP-1059-SOSIP followed by a boost of soluble 1059-SOSIP (Fig. 2). Rabbits were divided into 2 groups with only one difference between the groups: Group 1 was boosted (first boost) with mRNA-LNPs for expression of HIV-1_AD8_ ΔCT Envs whereas Group 2 was boosted (first boost) with mRNA-LNPs for co-expression of HIV-1_AD8_ ΔCT Envs and HIV-1 Gag in target cells. mRNA-mediated co-expressing HIV-1_AD8_ ΔCT Envs and HIV-1 Gag in the same cell produces viral-like particles displaying the ΔCT HIV-1_AD8_ Envs on their surfaces in vitro (Fig. 1d). We collected blood at specified intervals (Fig. 2a) and analyzed the serum for the antibody response. All rabbits developed high-affinity antibodies over the time of immunization, with high titer (>1:10,000) against the 1059-SOSIP at week 36 (Fig. 2b, c). The median titer of all sera increased by 58.8-fold after the synVLP boost (median ID_50_ 1:74 vs 1:4350) and further by 6.6-fold after the soluble 1059-SOSIP boost (median ID_50_ 1: 4350 vs 1:28,738). Serum antibodies from all rabbits at week 36 bound to 1059-SOSIP at comparable levels to their binding to 1059 gp120 (Fig. 2c, d), suggesting that (a) most antibody responses were directed against gp120, and (b) antibodies did not target a “glycan hole” in the SOSIP trimer that is a dominant immunogen observed for other SOSIP immunizations but absent in the gp120 monomer. Binding antibody response was broad as judged by sera binding to the heterologous BG505, CH040, and NAB9_Q658V_ SOSIPs and to the homologous AD8 gp120, although the titer in most of these cases was significantly lower compared with the titer for binding to the 1059 Envs (SOSIP or gp120). Notably, we identified a significant difference between the two groups of rabbits: sera from group 1 exhibited significantly stronger Env binding (higher binding titer 50) compared to sera from group 2 (Fig. 2f). We also detected a higher average number of antibody-secreting cells (ASC) in group 1 compared to group 2 (Fig. 2g), but this difference did not reach a statistically significant value (Fig. 2h), mainly because rabbit 5 in group 2 showed an exceptionally high level of ASC in the blood.

**Fig. 3:**
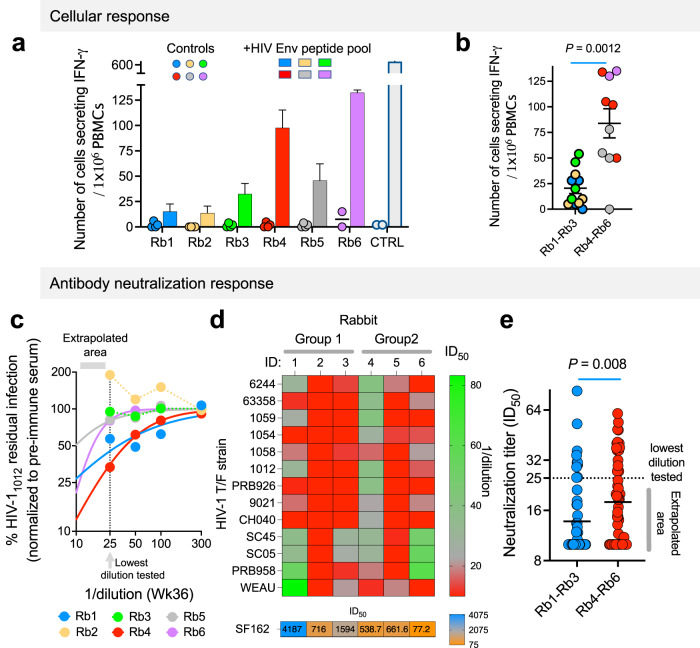
Cellular and neutralizing antibody responses to conformation-specific HIV-1 Env immunogens delivered by mRNA-LNP and syn-VLPs to rabbits. **a** Response of PBMCs (collected 42 weeks post priming) to incubation with HIV-1 Env peptide pool was detected by ELISpot assay. Four measurements (controls) or the mean ± sem are shown for each rabbit. Results of rabbit 6 PBMCs are from only 2 measurements due to limited cells availability. Controls are PBMCs incubated with medium without peptide pools. CTRL, positive control of pooled PBMCs stimulated with PMA/Ionomycin (2 measurements). **b** Statistical analysis of the difference between the number of PBMCs secreting IFN-γ in response to HIV-1 Env peptides (from panel a) in the 2 groups of rabbits. Group 1 was boosted (1st boost) with mRNA-LNPs for AD8ΔCT Env expression and Group 2 was boosted with mRNA-LNPs for co-expression of AD8ΔCT Envs and HIV-1 Gag, potentially leading to presentation of ΔCT Envs on virus-like particles in vivo. Color code is similar to panel (**a**) and represents 4 measurements (2 measurements for Rb6 and control) for PBMCs from each rabbit. **c** Neutralization activity of sera from the 6 rabbits against viruses pseudotyped with HIV-1_1012_ Envs. Dash line indicates the effect of lowest dilution of sera tested (1:25) from each rabbit on viral entry; the area below the lowest dilution is labeled as extrapolated area to indicate that in some cases of very weak response, ID_50_ values were calculated from fitted curves and could not have been tested experimentally. **d** Neutralization titer (Inhibitory dilution 50; ID_50_) of rabbit sera from week 36 post immunization against 13 T/F strains and against the easy-to-neutralize HIV-1_SF162_ strain (pseudoviruses). ID_50_ values are color-coded according to the color scale shown on the right. ID_50_ < 10 was set to the value 10. Exact ID_50_ values of the sensitivity of HIV-1_SF162_ strain to each rabbit sera are indicated in the lower panel. **e** statistical analysis of the difference between neutralization titer of the 2 groups of rabbits (groups specified in Fig. 2a). Lowest dilution of sera tested (1:25) and values calculated from extrapolated fit below 1:25 dilutions are indicated. *P*, two-tailed *P* value of Mann–Whitney U-test. Neutralization results are representative of between 1 and 3 independent experiments, each performed in duplicate. Mean ± sem / individual points (**a**, **b**) or geometric mean (**e**) of all samples are shown.

We evaluated the frequency of PBMCs that were specific for HIV-1 Envs by measuring the number of cells that were activated by a pool of HIV-1 Env peptides (ELISpot). PBMCs from all 3 rabbits of group 2 contained a higher number of HIV-1 Env-specific cells than PBMCs from any of the rabbits in group 1, and the difference between the 2 groups was statistically significant (Fig. 3a, b). Moreover, we identified two patterns of reactivity: group 1 showed high binding to soluble Envs but low cellular anti-Env response whereas group 2 exhibited the reverse phenotype with relatively lower binding to soluble Envs but high cellular anti-Env response. To assess the contribution of the cellular response to the development of a neutralization response to HIV-1, we measured the ID_50_ of all rabbit sera to viruses pseudotyped with different Envs. Sera from all 6 rabbits efficiently neutralized the easy-to-neutralize HIV-1_SF162_ pseudoviruses at high titer (ID_50_ > 1:2000 at week 36; Fig. 3d). None of the sera neutralized any of the 13 T/F entry very efficiently but sera from a subset of rabbits exhibited low titer but broad neutralization activity. In all our experiments, we used the pre-immune serum of each specific rabbit at each specific dilution to control for non-specific effects of serum on our neutralization assay (Supplementary Fig. 7). We could fit most serum dose–response curves to the logistic equation and in some cases of very weak response extrapolated any ID_50_ values that were below 1:25 dilution and could not have been tested experimentally due to non-specific effects of serum on our assay (Supplementary Fig. 7). Low-titer serum of rabbit 4 (Rb4) neutralized 11 out of 13 T/F strains with a geometric mean of 1:27.2 over all 13 T/F Envs. We did not find any correlation between sera binding and efficient neutralization; sera of Rb1 exhibited the strongest binding (lower dilution) and sera of Rb4 and Rb6 exhibited the lowest binding whereas all 3 sera showed some low-titer neutralization activity. In contrast, Sera of Rb2, Rb3, and R5 exhibited intermediate binding among the 6 sera tested but showed no, or almost no, neutralization effects. Importantly, we did identify a strong association between the frequency of specific anti-Env PBMCs and viral neutralization activity. Global analysis of the sensitivity of all 13 T/F strains to rabbit sera from each group, which included in some cases extrapolated ID_50_ values, showed that inclusion of HIV-1 Gag in Group 2 (Rb4-Rb6) was beneficial and resulted in broader neutralization and statistically significant higher neutralization titer (Fig. 3d, e; *P* = 0.008).

Development of an effective HIV-1 vaccine is extremely challenging but significant progress has been made in recent years due to incremental efforts and guiding insights. Here we show that a sequential immunization scheme with conformationally diverse Envs results in low titer but broad neutralization activity in a subset of rabbits. One out of two rabbits in the preliminary study and three out of the six rabbits in the subsequent study developed a low-titer anti-viral antibody response. Importantly, we identified a potential trigger for the elicitation of neutralization response that is associated with a high cellular response but not with high levels of sera binding to soluble Envs. mRNA-mediated co-expression of HIV-1_AD8_ ΔCT Envs and HIV-1 Gag, which probably generated VLPs presenting HIV-1_AD8_ ΔCT Envs in vivo, induced a weak but broad neutralization response. It is unlikely that mRNA-mediated HIV-1 Gag expression by itself could be responsible for this effect as all rabbits were immunized (second boost; third immunization) with mRNA for co-expression of HIV-1 Gag with a full-length Envs from a strain other than HIV-1_AD8_. Rather, the exact combination of mRNA-based, and co-expression of HIV-1_AD8_ ΔCT Envs and HIV-1 Gag were required to enhance the neutralization response. HIV-1_AD8_ Envs were isolated from a macrophage/T cell tropic strain (infects both macrophages and T cells), are very stable, and are expressed at high levels on the cell surface. In addition, Rhesus Macaques can be readily infected with SHIV-AD8 (simian Immunodeficiency virus carrying the AD8 *env* gene) and this knowledge provides future opportunities to potentially test any vaccine candidates in HIV-1 (SHIV) challenge experiments in monkeys.

**Fig. 4:**
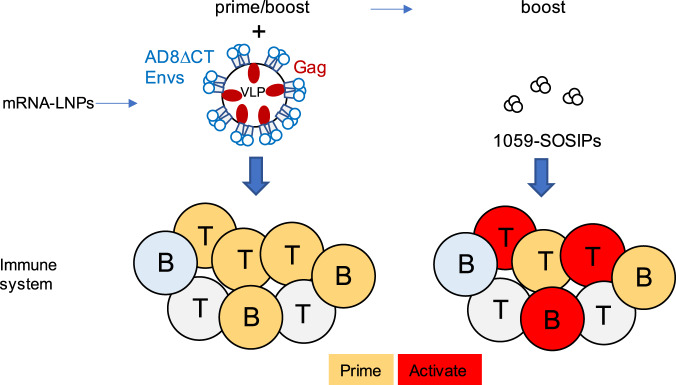
A model for triggers of enhanced neutralization response in rabbits. Immunization with mRNA for co-expressing AD8ΔCT Envs and HIV-1 Gag, which potentially leads to production of VLPs displaying the AD8ΔCT Envs in vivo, can prime diverse T and B cells. Some of these cells can be then activated by immunization with soluble 1059-SOSIP. The overall result is enhanced neutralization and cellular response. ΔCT cytoplasmic tail-deleted.

Typically, ΔCT Env variants are expressed on the surface of cells at 10-fold higher levels than full-length WT Envs, and specifically, HIV-1_AD8_ ΔCT Envs on pseudoviruses resulted in viruses that were significantly more infectious than HIV-1_AD8_ WT pseudoviruses (Fig. 1b). Thus, we hypothesize that high levels of entry-compatible Envs displayed on VLPs presented the Env immunogen in an optimal way to the immune system; and combined with the mRNA platform triggered the development of an enhanced neutralizing response, which was associated with a high frequency of anti-Envs PBMCs (Fig. 4). Overall, we could identify a distinct pattern of humoral immune response in each of the two groups of rabbits: the antibody response in group 1 was channeled toward high binding antibodies to soluble Envs whereas the response in group 2 efficiently activated a cellular anti-Env response and elicited neutralizing antibodies without the requirement to elicit high binding to soluble Envs. This approach may be further used to manipulate the type of antibody elicited by specific vaccination.

Our study expands the knowledge about, and provides new insights into, the development of an effective HIV-1 vaccine. Major limitations of our conclusions are related to the elicitation of neutralization response in only a subset of rabbits and the low levels of neutralization. However, with these limitations we could detect significant difference between the groups, mainly because only 1 out of 3 rabbits in group 1 but 2 out of 3 rabbits in group 2 showed low-titer broad neutralization activity. Thus, challenges to our tested approach will be to develop ways to increase the potency of this response without compromising breadth and to increase the frequency or robustness of such response.

## Methods

### Plasmid construction

All plasmids for soluble SOSIP Env expression were codon-optimized and included the tissue plasminogen activator (TPA) signal peptide instead of the natural signal peptide. A SpyTag SOSIP Env-expressing plasmid was generated by adding downstream to the 1059-SOSIP gene (after residue 664) the DNA sequences encoding for the following peptides: an 8-reside linker sequence (GSGSGGSG), an 8-residue polyhistidine tag (HHHHHHHH), and a 13-residue SpyTag peptide (AHIVMVDAYKPTK). DNA was cloned by Gene Universal (Newark, DE). BG505 SOSIP.v6 expression plasmid was a kind gift from R.W. Sanders and I. Del Moral Sanchez (University of Amsterdam) and supports the expression of soluble, stabilized SOSIP.v6 trimer that contains additional 8 amino acid changes in comparison with BG505 SOSIP^28^. SpyCatcher003-mi3 plasmid was from Addgene (Cat# 159995). In vitro transcription (IVT) cassettes for mRNA synthesis were designed in-house and included bacteriophage T7 RNA polymerase promoter, stable untranslated regions (UTRs) based on previously published reports^29–31^, gene-of-interest, and a 120–125 base polyA tail (Supplementary Fig. 4). Designed DNA was synthesized and cloned into a pUC19 plasmid by Gene Universal. All plasmids were fully sequenced to verify correct sequence of the IVT cassettes. IVT plasmid for mRNA-mediated expression of HIV-1_AD8_ ΔCT Envs was generated by digesting the parental HIV-1_AD8_ WT env plasmid with BsiWI restriction enzyme (2 sites) to remove the CT DNA-encoding fragment and self-ligating the digested vector. The resulting vector contained only a 27-base polyA tail due to bacteria-mediated deletion during generation of this variant.

### Protein expression and purification

293 F cells were co-transfected with a SOSIP- or SpyTag SOSIP-expressing plasmid and a human furin-expressing plasmid at a ratio of 4:1 using Turbo293 transfection reagent (Speed Biosystems; Gaithersburg, MD). Transfected cells were grown on a shaker in a tissue culture incubator at 37 °C, 8% CO_2_ for 3–5 days, and culture supernatants were then harvested, clarified by centrifugation at 4000 × *g* for 20 min, and filtered through 0.2 μm filter (VWR). Supernatant-containing SOSIP glycoproteins was loaded on a *Galanthus nivalis* lectin (GNL) column (Vector Laboratories) at 4–8 °C, the column was washed with 500 mM NaCl in phosphate-buffered saline pH 8 and proteins were eluted with 1 M Methyl-α-D-mannopyranoside/PBS solution, filtered through 0.2 μm filter and concentrated using Vivaspin 6 centrifugal concentrators (30 kDa; Cytiva). Purified SOSIP glycoproteins were then separated on a HiLoad 16/600 Superdex 200 pg size-exclusion chromatography column (Cytiva) and SOSIP trimer fractions were pooled, concentrated, and stored in aliquots at −80 °C until use. Full and un-cropped scans of the SDS-PAGE gels and western blots are provided as Supplementary Fig. 8.

### SpyCatcher003-mi3 protein expression and purification

Synthetic nanocages displaying SpyCatcher on their surface were expressed and purified following protocols adapted from Rahikainen et al.^25^ Briefly, SpyCatcher003-mi3 plasmid was transformed to E. coli BL21(DE3) RIPL bacteria (Agilent, Santa Clara, CA) and transformed bacteria were grown on LB-Agar plates supplemented with 50 µg/mL kanamycin for 16 h at 37 °C. Subsequently, a single colony was picked and grown in a 10 mL LB culture containing 50 µg/mL kanamycin at 37 °C for 16 h with continuous shaking. The culture was diluted 1:100 into LB containing 50 µg/mL kanamycin and 0.8% (w/v) glucose and incubated at 37 °C with continuous shaking until OD_600_ reached 0.6. The bacteria were then induced with 0.42 M Isopropyl ß-D-1-thiogalactopyranoside (IPTG) (Fisher Scientific) at 22 °C for 16 h, centrifuged, and suspended in 25 mM Tris–HCl, 300 mM NaCl, pH 8.5. The bacteria were lysed by sonication (4 times for 60 s, at a 50% duty-cycle, using an ultrasonic processor (Qsonica) equipped with a microtip) in the presence of 0.1 mg/mL lysozyme (Sigma), 1 mg/mL cOmplete mini EDTA-free protease inhibitor (Merck), and 1 mM phenylmethanesulfonyl fluoride (PMSF) (Thermo Scientific). The lysate was clarified by centrifugation at 35,000 × g for 45 min at 4 °C, filtered through a 0.22 µm filter, and treated with 170 mg ammonium sulfate per mL of lysate followed by incubation at 4 °C for 1 h with continues mixing using with a magnetic stirrer. The precipitated nanocages were collected by centrifugation at 30,000 × *g* for 30 min at 4 °C, resuspended in 25 mM Tris–HCl, 150 mM NaCl, pH 8.5, and then dialyzed against 500-fold excess of the same buffer to remove residual ammonium sulfate. The SpyCatcher003-mi3 nanocages were further clarified by centrifuging at 17,000 × *g* for 30 mins at 4 °C, filtered to remove any insoluble material, and purified by size-exclusion chromatography (SEC) using a HiPrep Sephacryl S-400 HR 16–600 SEC column (GE Healthcare) equilibrated with 25 mM Tris–HCl, 150 mM NaCl, pH 8.5. Fractions containing the SpyCatcher003-mi3 nanocages were identified and concentrated using a Vivaspin 20 100 kDa MWCO concentrator. Endotoxin was removed from the sample using Triton X-114 (Sigma) phase separation as previously described^32^. Briefly, 1% (v/v) Triton X-114 was mixed with SpyCatcher003-mi3 by gentle pipetting and incubated on ice for 15 min to dissolve all the Triton X-114. The sample was then incubated in a 37 °C water bath for 5 min, centrifuged at 16,900 × *g* for 5 min at 30 °C, and the top phase was carefully transferred into a new microcentrifuge tube and the method was repeated twice. The final concentration of the endotoxin-depleted purified particles was measured by bicinchoninic acid (BCA) assay (Pierce, ThermoFisher Scientific) and nanocages purity was assessed by SDS-PAGE.

### 1059-SOSIP display on viral-like particles (synthetic nanocages)

1059-SOSIP-SpyTag and SpyCatcher nanocages were conjugated by incubation at different ratio (between 1:1 and 6:1) in 25 mM Tris–HCl, 150 mM NaCl, pH 8.0 at 4 °C for 16 h. Aggregates were removed by centrifugation at 16,900 × *g* for 30 mins at 4 °C. To confirm conjugation, samples were separated on an 8–16% Mini PROTEAN TGX stain-free gradient gel (Bio-Rad) under reducing conditions and imaged using a ChemiDoc XRS+ imager (Bio-Rad). At 6:1 ratio (SOSIP: synthetic nanocages) most of the nanocages were saturated and the unbound SOSIP Env was removed by SEC.

### ELISA

We used enzyme-linked immunosorbent assay (ELISA) to analyze rabbit sera binding to SOSIP trimers. GNL was immobilized in a 96-well plate (Greiner Bio-One, NC) by adding 0.2 μg of GNL in 100 μl PBS in each well and incubating the plates overnight at room temperature (RT). Next, the wells were washed 3 times with PBS containing 0.2% Tween-20 (wash solution) using an in-house vacuum system and blocked with PBS containing 3% bovine serum albumin (blocking solution) for 2 h at RT. The wells were then washed 3 times, 0.2–0.25 μg of purified SOSIP trimers in blocking solution were added to test wells, and the plate was incubated for 1–2 h(s) at RT. Wells were washed 6 times and different dilutions of rabbit sera in a blocking solution were added. After 1 h and 30 min incubation, wells were washed 6 times, and 1:50,000 dilution of Peroxidase AffiniPure F(ab’)_2_ Fragment Goat Anti-Rabbit IgG (H + L) (cat# 111–036–045; Jackson ImmunoResearch, PA) was added in blocking solution to each well and the plate was incubated for 1 h at RT. Wells were then washed 6 times and 100 μl of TMB solution (1 ml of 1 mg/ml 3,3,5,5-tetramethylbenzidine (Sigma) in DMSO, 9 ml of 0.1 M sodium acetate, pH 5.0, and 2 μl of fresh 30% hydrogen peroxide) was added to each well. After ~18-min incubation, the HRP reaction was stopped by adding 50 μl of 0.5 M H_2_SO_4,_ and optical density at 450 nm was measured using a spectrophotometer. We used the same procedure to measure the binding of human antibodies to the 1059-SOSIP. The following primary human antibodies were obtained from the NIH HIV Regent Program and used at 0.625 µg/ml: PGT121 (cat# ARP-12343), PG9 (cat# ARP-12149), VRC03 (cat# ARP-12032), F105 (cat# ARP-857), 2G12(cat# ARP-1476), VRC01 (cat# ARP-12033), 39 F (cat# ARP-11437), PGT145 (cat# ARP-12703), 10-1074 (cat# ARP-12477), and 17b (cat# ARP-4091). We used HRP-conjugated donkey anti-human IgG secondary antibodies at 1:5,000 dilution for detection (Peroxidase AffiniPure F(ab’)_2_ Fragment Donkey Anti-Human IgG, Fcγ fragment specific; cat# 709-036-098; Jackson ImmunoResearch Inc.).

### In vitro transcription, mRNA purification & mRNA-LNP preparation

mRNA was simultaneously in vitro transcribed and capped using the T7-FlashScribe transcription kit (CellScript, Madison, WI) and CleanCap reagent AG (TriLink Biotechnologies, San Diego, CA) according to the manufacturer’s instructions. In some cases, we used modified pseudouridine (N1-methyl-pseudouridine-5′-triphosphate) instead of uridine-5′-triphosphate to stabilize the mRNA molecules. Transcribed mRNA was purified using a MEGAclear transcription clean-up kit (ThermoFisher Scientific) according to the manufacturer’s instructions and impurities were further removed on cellulose columns as previously described^33^. We measured mRNA concentration by optical density at 260 nm and froze the mRNA preparation at −80 °C until it was encapsulated by LNPs at Acuitas Therapeutics Inc (Vancouver, British Columbia, Canada). All mRNAs used for mRNA-LNP preparation were transcribed in vitro using modified pseudouridine and IF4-based plasmids (pIF4; Supplementary Fig. 4) except for second immunization of rabbits group 1, in which mRNA-LNPs contained mRNAs encoding for HIV-1AD8 ΔCT that were separately transcribed from two plasmids: IF4 and TEV (50% from pIF4 and 50% from pTEV; Supplementary Fig. 4). For co-expression and VLP generation, mRNA encoding HIV-1 Envs was mixed with mRNA encoding for HIV-1_NL4-3_ Gag at 1:1 ratio (based on experiments shown in Fig. 1d) and the total mRNA molecules were encapsulated in LNPs. All mRNA-LNP were stored in single-use tubes at −80 °C.

### Animal care

Experiments involving New Zealand White rabbits were carried out according to NIH guidelines for the housing and care of laboratory animals. Protocols were reviewed and approved by the Institutional Animal Care and Use Committee (IACUC) of the University of Minnesota.

### Rabbit immunizations

Rabbits were intramuscularly immunized in a single site in the quadriceps muscle with either mRNA-LNPs (35 μg/rabbit), synVLP-1059-SOSIP (21.5 μg/rabbit) or soluble SOSIP trimers (50 μg/rabbit). Protein immunizations were administered after mixing the protein solution (PBS) with AddaVax adjuvant (InvivoGen, San Diego, CA) at a 1:1 volume ratio. Due to technical error, during the first immunization with mRNA-LNPs, rabbit 6 was immunized with only 11 μg and an additional immunization with 24 μg was administrated one week later. Experiment end points were week 37 (preliminary experiment 1) and week 36 (experiment 2) post the first immunizations in each experiment.

### Blood collection

Blood was collected from the marginal ear vein/central ear artery according to an approved IACUC protocol. No anesthesia was used during blood collection. Rabbits’ ears were rubbed with gauze containing wintergreen oil before blood collection.

### Rabbit anesthesia and euthanasia

Rabbits were euthanized by professional team at the animal facilities of the University of Minnesota under the supervision of a certified veterinarian and according to the approved IACUC protocol. Pentobarbital was used for euthanasia of the rabbits, and the following drugs were used for sedation prior to euthanasia: Ketamine 35 mg/kg IM; Xylazine 5 mg/kg IM; and Acepromazine 1 mg/kg IM. After sedation, 1.5 ml/5 kg Euthasol (pentobarbital sodium and phenytoin sodium) was administered IV to euthanize the rabbits. Euthanized rabbits were subjected to exsanguination according to the approved IACUC protocol.

On one occasion, due to ketamine shortage, the veterinarians recommended the temporary use of additional drug options for sedation prior to euthanasia for a subset of rabbits. These included Torbugesic (butorphanol tartrate) 0.5 mg/kg IM, midazolam 1.0 mg/kg IM, and dexmedetomidine 0.01 mg/kg IM.

### Production of single-round pseudoviruses

We produced pseudoviruses as we previously described^9^^,15,34–36^^.^ Briefly, a packaging plasmid (psPAX2), a reporter plasmid (pHIVec2.luc), and an Env-expressing plasmid were co-transfected into 293 T cells using effectene transfection reagent (Qiagen). After a 48-h incubation, the cell supernatant was collected and centrifuged for 5 min at 600–900 × *g* at 4 °C. The amount of p24 in the supernatant was measured using the HIV-1 p24 antigen capture assay (Cat# 5421, Advanced BioScience Laboratories), and the virus-containing supernatant was frozen in single-use aliquots at −80 °C. In some cases, the Env-expressing plasmid was replaced by Env-expressing mRNA that was transfected using Trans-IT (Mirus Bio LLC, Madison, WI) or by the direct addition of mRNA-LNPs, both 24 h post-transfection of the packaging and reporter DNA plasmids.

### Viral infection assay

A single-round infection assay was performed in 96-well white plates (Greiner Bio-One, NC) using TZM-bl target cells (NIH AIDS Reagent Program) as previously described^37^. Thirty μl of diluted serum was added to each well followed by the addition of 30 μl of specified pseudoviruses and the plate was incubated for 1-h at 37 °C in a tissue culture incubator with 5% CO_2_. Then, approximately 7000 TZM-bl cells in 30 µl DMEM were added to each well, the plate was incubated for 48 h, cell lysed, and luciferase activity was measured by Centro XS³ LB 960 microplate luminometer (Berthold Technologies GmbH & Co. KG, Germany). Dose–response curves were non-linearly fitted to the logistic equation using Prism 9 (GraphPad Software, Boston, MA), after importing the logistic equation to the analysis software, as previously described^34,38–41^ but doses were expressed as dilution instead of absolute concentration and reported parameters were half-maximal inhibitory dilution (ID_50_). Lowest dilution tested was 1:20 for experiment 1 or 1:25 for experiment 2 (Supplementary Table 1). Due to weak neutralization response, ID_50_ values in some cases were below the lowest dilution tested (non-specific effects of the serum prevented testing lower than 1:25 dilution for experiment 2) and they were based on extrapolation of the fitted curves.

### Peripheral blood mononuclear cells (PBMCs) isolation

Blood was collected from the ear vein of rabbits in 6 ml heparinized tubes and used immediately after collection. PBMCs were purified by density centrifugation (800 × *g* for 30 min) on Lymphocyte separation medium 1077 (PromoCell, Heidelberg, Germany), isolated from the gradient interface, washed twice in Dulbecco’s phosphate-buffered saline (DPBS, ThermoFisher Scientific, Waltham, MA), and resuspended in Roswell Park Memorial Institute (RPMI) 1640 medium (ThermoFisher Scientific, Waltham, MA) supplemented with 10% heat-inactivated fetal bovine serum (FBS).

### ELISpot assay

ELISpot assay was based on the ELISpot Flex: Rabbit IFN-γ (HRP) kit (Mabtech, Cincinnati, OH). On day 1, a 96-well PVDF membrane white plate (Mabtech, Cincinnati, OH) was pre-treated with 35% ethanol for 1 min to activate the membrane and obtain maximal antibody binding capacity. The plates were then coated with 100 µl of 15 µg/ml unconjugated anti-rabbit IFN-γ mAb (MT327) in PBS and incubated overnight at 4 °C. On day 2, the plate was washed 5 times with sterile PBS and then blocked with RPMI 1640 medium at room temperature for at least 30 min. Freshly isolated PBMCs were added to the plate together with 2 µg/ml of the Peptide Pool, HIV-1 Subtype B (Consensus) Env Region (NIH-ARP Cat# 12540). Medium-only samples were added as controls to assess background level of lymphokine secretion. PMA/ionomycin was added to positive control wells. The plate was incubated for 18 h at 37 °C in 5% CO_2_ tissue culture incubator. On day 3, the plate was washed with PBS and then incubated for 2 h at room temperature with 0.1 µg/ml of biotinylated anti-rabbit IFN-γ mAb (MT318). The plate was then washed and incubated with 100ul of Streptavidin-HRP (1:1000 dilution) for 1 h at room temperature. ELISpot substrate (TMB; Mabtech, Cincinnati, OH) was added until distinct spots appeared. The plate was dried at room temperature overnight. On day 4, the number of spots in each well was measured using the CTL Immunospot analyzer (Cleveland, OH). We used 2 different cell densities in duplicate (either 2*10^5^ or 5*10^5^ cells/well), normalized the results to the frequency of cells in 1*10^6^ PBMCs and averaged the 4 replicates (2 from each cell density).

### ELISpot IgG assay

ELISpot IgG assay was based on the ELISpot Flex: Rabbit IgG (HRP) kit (Mabtech, Cincinnati, OH). On day 1, a 96-well PVDF membrane white plate (Mabtech, Cincinnati, OH) was pre-treated with 35% ethanol for 1 min to activate the membrane and allow maximal antibody binding capacity. The plate was washed 5 times with sterile water and coated with 100 µl of 2.5 µg/ml unconjugated antigen (1059-SOSIP trimer) in PBS and incubated overnight at 4 °C. On day 2, the plate was washed 5 times with sterile PBS and then blocked with RPMI 1640 medium with 10% FBS at room temperature for at least 30 min. Freshly isolated PBMCs suspended in RPMI 1640 10% FBS were added to the plate and medium-only samples were added as controls to assess the background level of IgG secretion. The plate was incubated for 20 h at 37 °C in 5% CO_2_ tissue culture incubator. On day 3, the plate was washed with PBS and then incubated for 2 h at room temperature with 0.5 µg/ml of biotinylated detection mAb (MT536; Mabtech, Cincinnati, OH) in PBS-0.5% FBS. The plate was then washed and incubated with 100 μl of Streptavidin-HRP (1:1000 dilution) for 1 h at room temperature. ELISpot substrate (TMB) was added until spots were visible. The plate was dried at room temperature overnight. On day 4, the number of spots in each well was measured using the CTL Immunospot analyzer (Cleveland, OH). We used 5*10^5^ cells/well in duplicate, subtracted background measurements of multiple wells (medium only) and calculated the number of antibody-secreting cells in 1*10^6^ PBMCs.

### Reporting summary

Further information on research design is available in the Nature Research Reporting Summary linked to this article.

### Supplementary information

Supplementary

Reporting summary

## Data Availability

Data are available in this paper, Supplementary information, or from the corresponding author upon request.
