# Supplementary material for: Enhancing anti-viral neutralization response to immunization with HIV-1 envelope glycoprotein immunogens
Source: NPJ Vaccines. 2023 Nov 24;8:181. doi: 10.1038/s41541-023-00774-z (PMC10667240; doi:10.1038/s41541-023-00774-z)
Supplement: Supplementary file 1 — Supplementary [file 41541_2023_774_MOESM1_ESM.pdf]

Enhancing anti-viral neutralization response to immunization with HIV-1 envelope glycoprotein immunogens

Shamim Ahmed, Durgadevi Parthasarathy, Rachael Newhall, Tashina Picard, Morgainne Aback, Sneha Ratnapriya, William Arndt, Widaliz Vega-Rodriguez, Natalie M. Kirk, Yuying Liang and Alon Herschhorn

Supplementary information

Supplementary Table 1. Sensitivity of T/F strains to serum of rabbits immunized with HIV-1 Envs according to the immunization scheme shown in Figures S1a (experiment 1) and 2a (experiment 2)[1]

**T/F Envs**	Experiment 1 (Rabbits A)[2]		Experiment 2 (Rabbits B)[3]					
			Group 1			Group 2		
	Ra1	Ra2	Rb1	Rb2	Rb3	Rb4	Rb5	Rb6
6244	ND	ND	30.0	Neg (<10)[4]	Neg (13.4)[4]	40.4	Neg (18.6)[4]	Neg (<10)[4]
63358	23.6	Neg (<10)[4]	Neg (11.8)[4]	Neg (<10)[4]	Neg (<10)[4]	36.7	Neg (<10)[4]	Neg (20.1)[4]
1059	67.5	Neg (16.4)[4]	25.3	Neg (<10)[4]	Neg (<10)[4]	41.1	Neg (<10)[4]	Neg (<10)[4]
1054	Neg (16.6)[4]	Neg (<10)[4]	Neg (<10)[4]	Neg (<10)[4]	Neg (10)[4]	27.5	Neg (13.2)[4]	Neg (14.7)[4]
1058	21.1	Neg (<10)[4]	Neg (18.3)[4]	Neg (<10)[4]	Neg (12.7)[4]	Neg (16.8)[4]	Neg (<10)[4]	Neg (21.75)[4]
1012	28.1	Neg (<10)[4]	31.1	Neg (<10)[4]	Neg (<10)[4]	38.1	Neg (<10)[4]	Neg (15.6)[4]
PRB926	Neg (19.6)[4]	Neg (10.1)[4]	Neg (10.8)[4]	Neg (<10)[4]	Neg (<10)[4]	37.8	Neg (<10)[4]	Neg (<10)[4]
9021	Neg (<10)[4]	Neg (<10)[4]	Neg (17.3)[4]	Neg (<10)[4]	Neg (<10)[4]	Neg (22.4)[4]	Neg (<10)[4]	Neg (19.5)[4]
CH040	Neg (19.6)[4]	Neg (13.0)[4]	Neg (<10)[4]	Neg (<10)[4]	Neg (<10)[4]	Neg (24.2)[4]	Neg (<10)[4]	Neg (<10)[4]
SC45	58.6	Neg (<10)[4]	36.4	Neg (<10)[4]	29.2	32.0	Neg (11.1)[4]	48.9
SC05	58.0	Neg (<10)[4]	34.3	Neg (<10)[4]	25.22	29.6	Neg (<10)[4]	54.1
PRB958	24.6	Neg (14.5)[4]	52.9	Neg (<10)[4]	Neg (11.9)[4]	Neg (18.7)[4]	Neg (<10)[4]	61.0
WEAU	30.8	Neg (<10)[4]	83.2	Neg (<10)[4]	Neg (21.3)[4]	Neg (11.6)[4]	Neg (19.3)[4]	Neg (<10)[4]

1 All neutralization assays were normalized to the effects of pre-immune serum at the same dilution of each specific rabbit. Values shown are inhibitory dilution 50 (ID_{50}). Titer values of experiment 2 are presented as a heat map in Figure 3d.

2 Week 25 sera

3 Week 36 sera

4 These values are below the lowest dilution tested (1:20 for experiment 1 or 1:25 for experiment 2) and they were calculated from the best non-linear fitted curve of all experimental points (different dilutions) to the logistic equation as described under the Methods section. Neg, Negative.

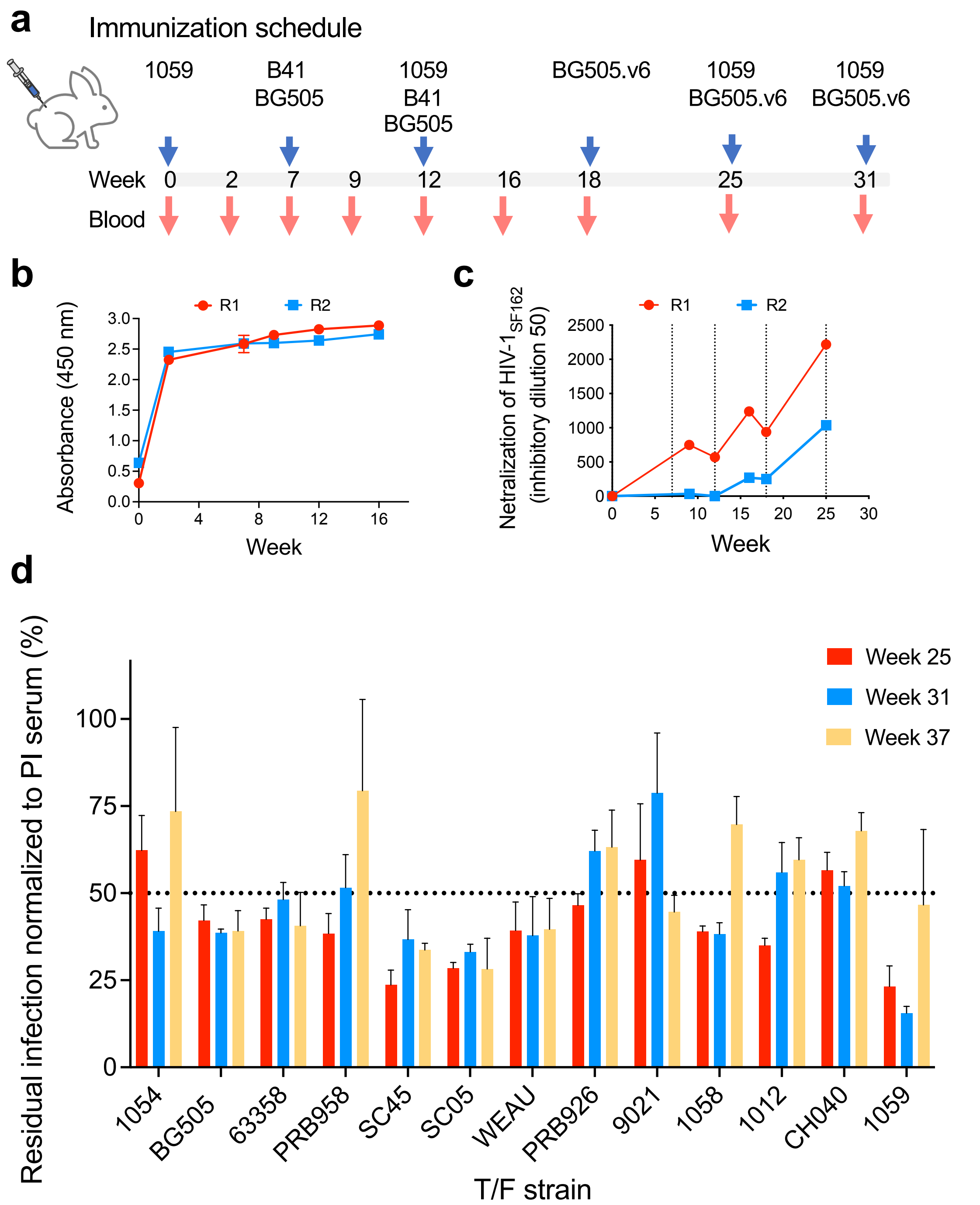

Supplementary Figure 1. Preliminary testing of conformation-specific HIV-1 Env immunogens. **a,** Immunization scheme of 2 rabbits with different soluble SOSIP-based HIV-1 Env trimers. 1059 Envs represent an incompletely closed SOSIP trimer of a clade B transmitted/founder (T/F) HIV-1 strain; BG505 and B41 are closed SOSIP Envs trimers of clade A and B HIV-1 strains, respectably. BG505 SOSIP.v6 contains several additional changes in comparison with BG505 SOSIP to stabilize the trimer and this version was used as it became available during the study. **b,** Binding of rabbit sera to immobilized 1059 SOSIP measured by ELISA. **c,** Sensitivity of viruses pseudotyped with the easy-to-neutralize (tier 1) HIV-1_{SF162} Envs to rabbit sera collected at specified time points (tested using Cf2-Th/$CD4^+CCR5^+$ target cells). **d,** Sensitivity of a panel of viruses pseudotyped with 13 T/F Envs (tier 2 and tier 3) to serum of rabbit 1 (R1) collected at weeks 25, 31 and 37 (1:20, 1:20, and 1:25 dilutions respectively). Sensitivity of this T/F panel to serum of R2 is shown in Fig. S3b. All viral neutralization values were normalized to the effect of pre-immune (PI) serum of the related rabbit at identical dilutions tested in the viral entry assay. Results are from one experiment performed in duplicate and mean ± range are shown.

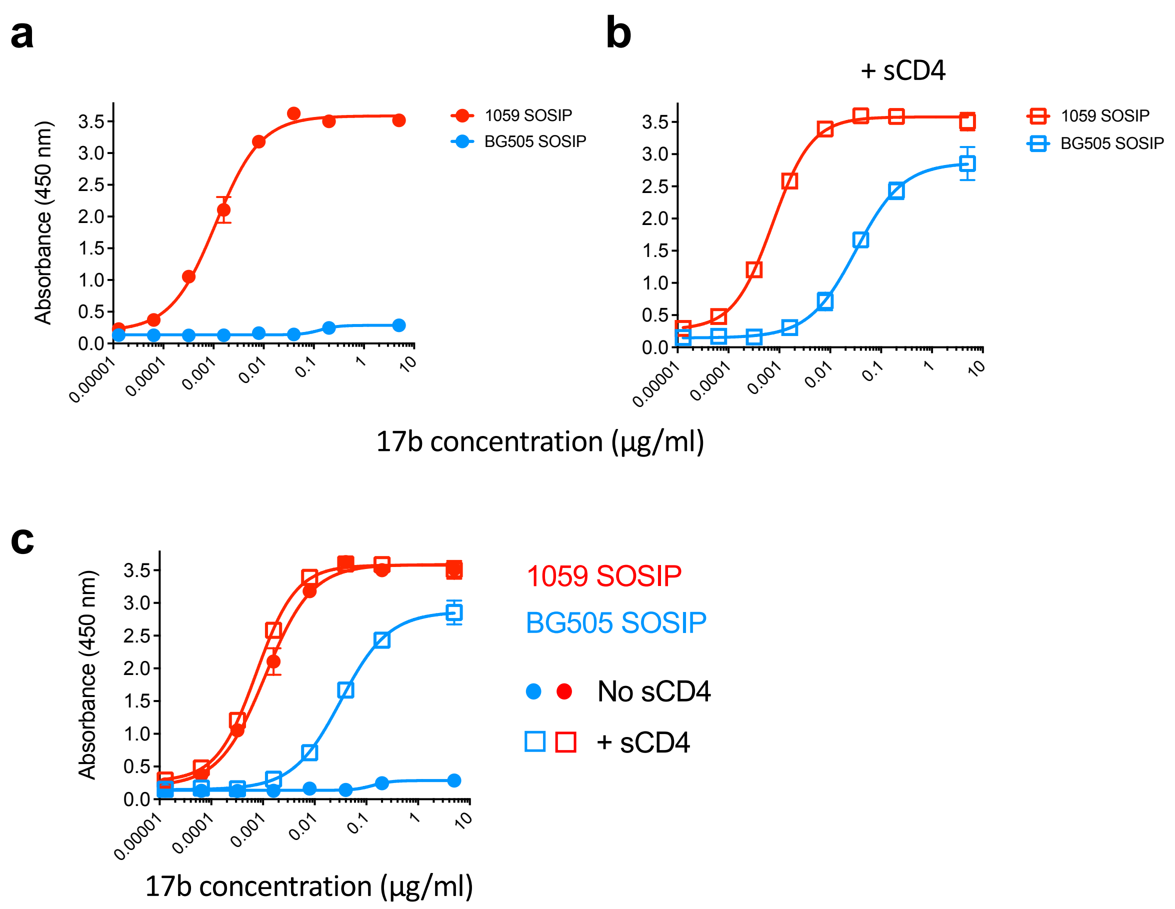

Supplementary Figure 2. Sensitivity of SOSIP trimers to 17b antibody in the presence and absence of soluble CD4 (sCD4). **a,** Dose response curves of the interactions of 17b antibody with BG505 and 1059 SOSIP trimers. The epitope of 17b overlaps with the coreceptor binding site and is typically occluded on the Envs of primary HIV-1 strains. **b,** Similar to (**a**) but in the presence of sCD4. **c,** Overlay of the curves in (**a**) and (**b**). A representative experiment performed in duplicate is shown (mean ± range).

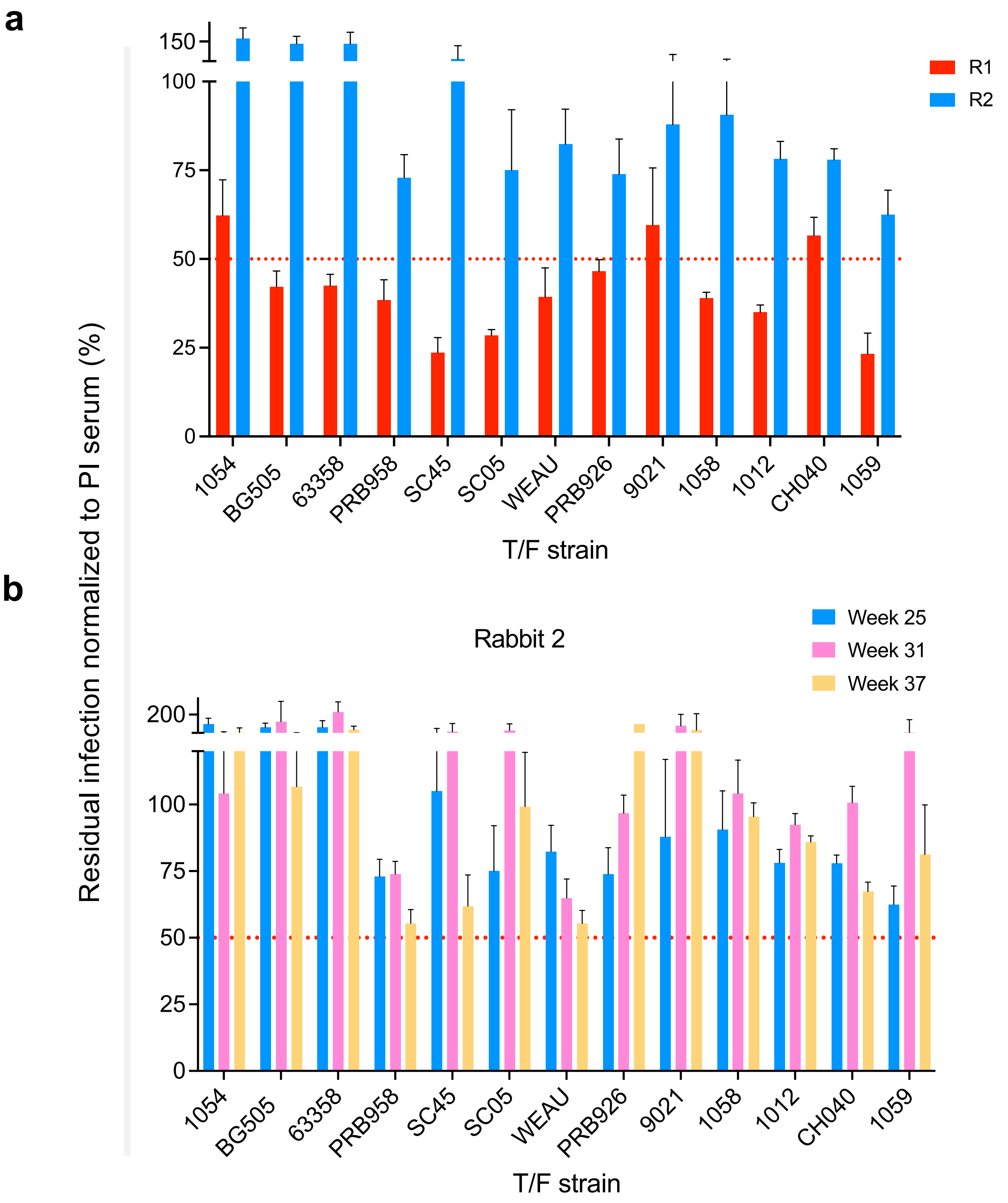

Supplementary Figure 3. Sensitivity of T/F strains to serum of rabbits immunized with Env at different conformations. Rabbits were immunized according to the scheme in figure 1 panel a and sera from blood collected at weeks 25, 31 and 37 was tested for neutralization of different T/F strains using TZM-bl target cells. **a,** Comparison of the neutralization effects of sera from rabbit 1 and of rabbit 2 (both collected at week 25; 1:20 dilution) on viral entry of viruses pseudotyped with 13 different T/F Envs. **b,** Effects of sera from rabbit 2 collected at weeks 25, 31, and 37 (1:20, 1:20, and 1:25 dilutions respectively) on viral entry of the same panel of pseudoviruses from (**a**). Results are from one experiment performed in duplicate and mean ± range are shown.

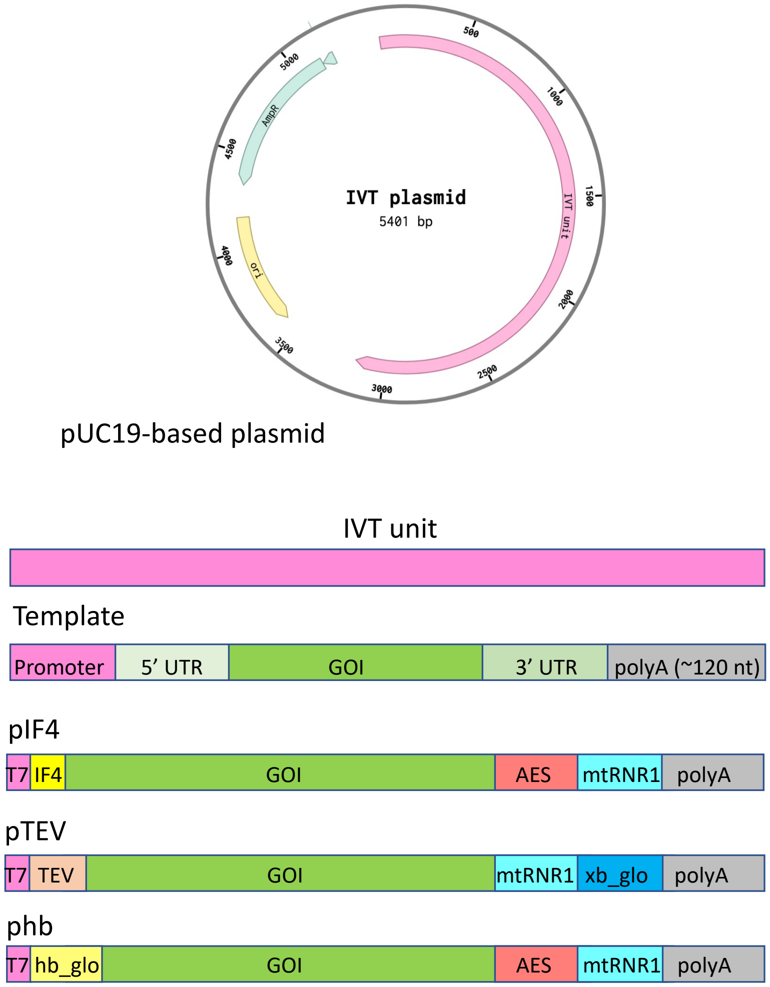

Supplementary Figure 4. In-vitro transcription (IVT) vectors. IVT plasmids were designed and built to contain bacteriophage T7 RNA polymerase promoter, 5' untranslated region (UTR), gene of interest, 3' UTR, and 120-base long polyA tail. IF4, 40-base long aptamer that binds eukaryotic translation initiation factor 4 G (Pascolo *et al.* 2019 CHIMIA); TEV, sequence derived from the 5' UTR of the tobacco etch virus (Gallie *et al.* 1995 Gene); AES/mtRNR1, 3'UTR stabilizing elements (Orlandini von Niessen *et al.* 2019, Molecular Therapy), xb_glo, a sequence derived from the 3'UTR of Xenopus beta-globin gene. GOI, gene of interest.

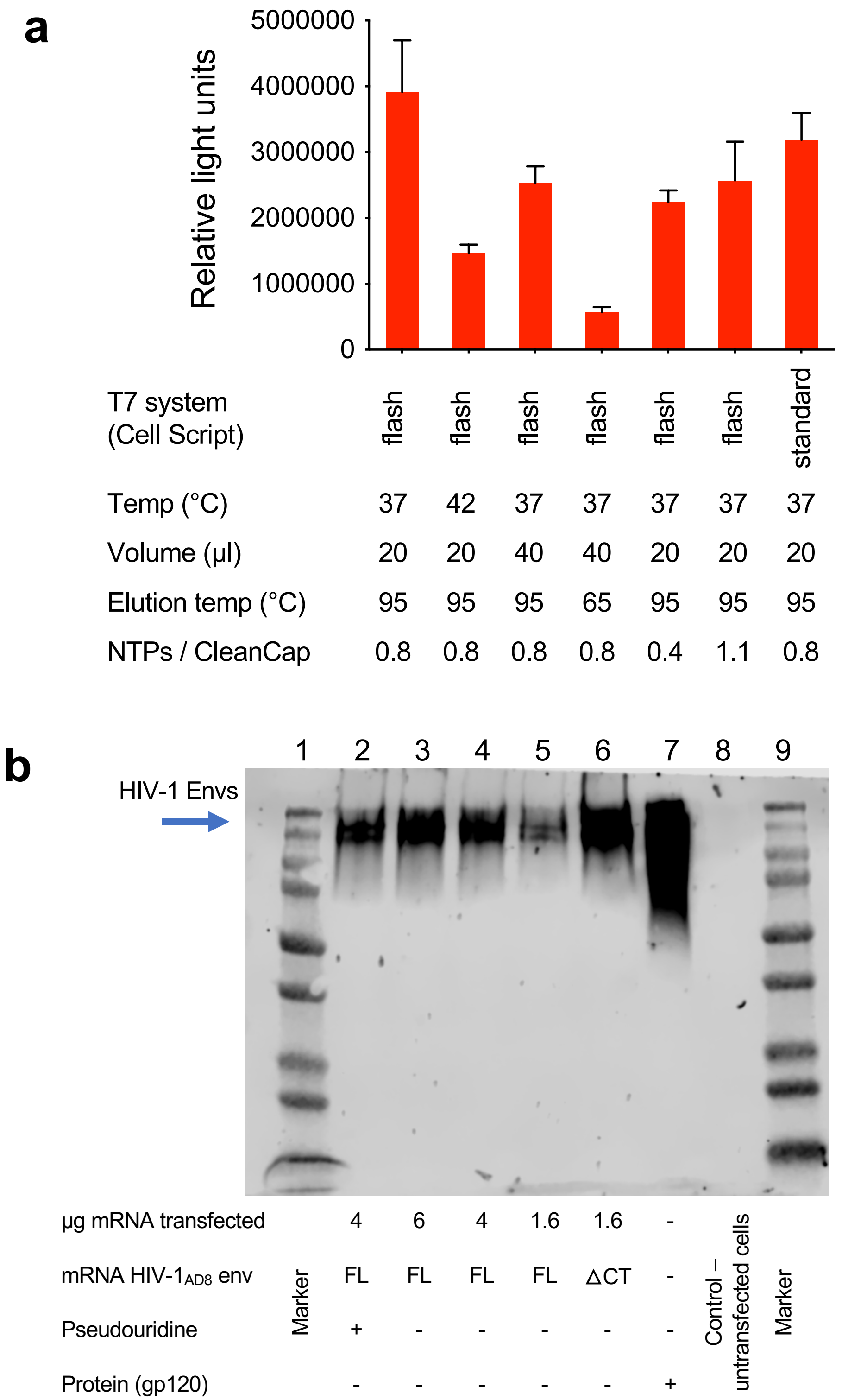

Supplementary Figure 5. Design and *in vitro* optimization of mRNA-based HIV-1 vaccine candidate. **a,** A vector for *in-vitro* transcription the firefly luciferase (fluc) mRNA was deigned, synthesized *de novo*, linearized and transcribed *in vitro* using the indicated conditions. We then purified the mRNA, transfected into 293T cells and measured the levels of fluc expression directly from lysed cells after 24 hours. Flash, T7-FlashScribe. Results are from one experiment performed in triplicate and the mean ± SD are shown. **b,** mRNA-mediated expression of HIV-1 Envs**.** Vectors for IVT of the indicated mRNAs were linearized, transcribed in vitro, purified and transfected into 293T cells, which were lyzed 24 hours later. HIV-1 Env expression in cell lysates was detected by western blot using serum from an HIV-1 infected Individual and secondary, HRP-conjugated anti human IgGs (1:10,000 dilution; Peroxidase AffiniPure F(ab')$_2$ Fragment Donkey Anti-Human IgG, Fcγ fragment specific; cat# 709-036-098; Jackson ImmunoResearch Inc.). All gels and blots were derived from the same experiment and were processed in parallel. FL, full-length; ΔCT, carboxyl-terminus deleted.

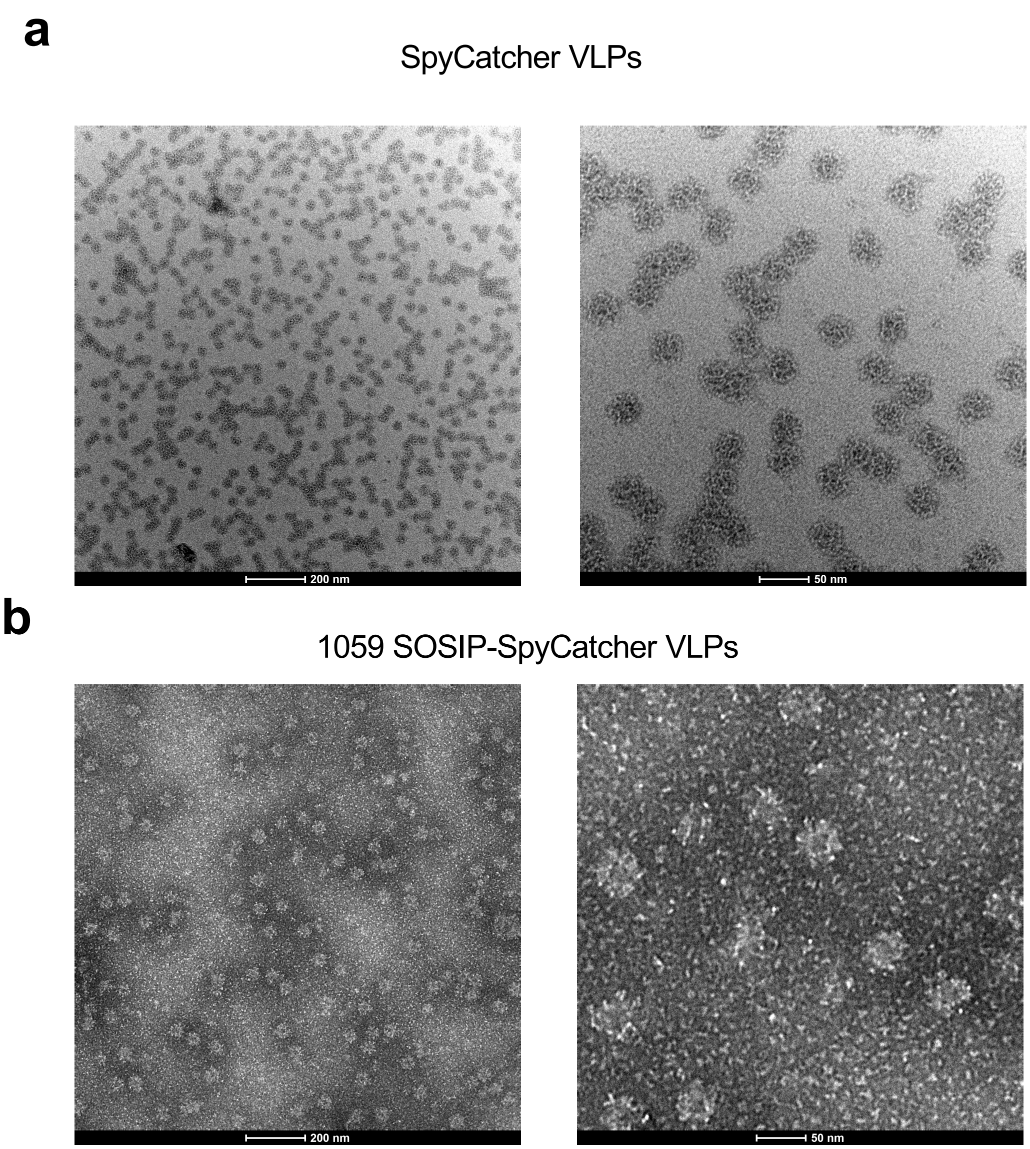

Supplementary Figure 6. Display of 1059-SOSIP on virus-like particles (VLPs) using SpyCatcher-based nanocages. **a,** Electron microscopy images of SpyCatcher-base nanocages (VLPs) after negative staining. **b,** Similar to panel a after conjugating the VLPs with 1059-SOSIP.

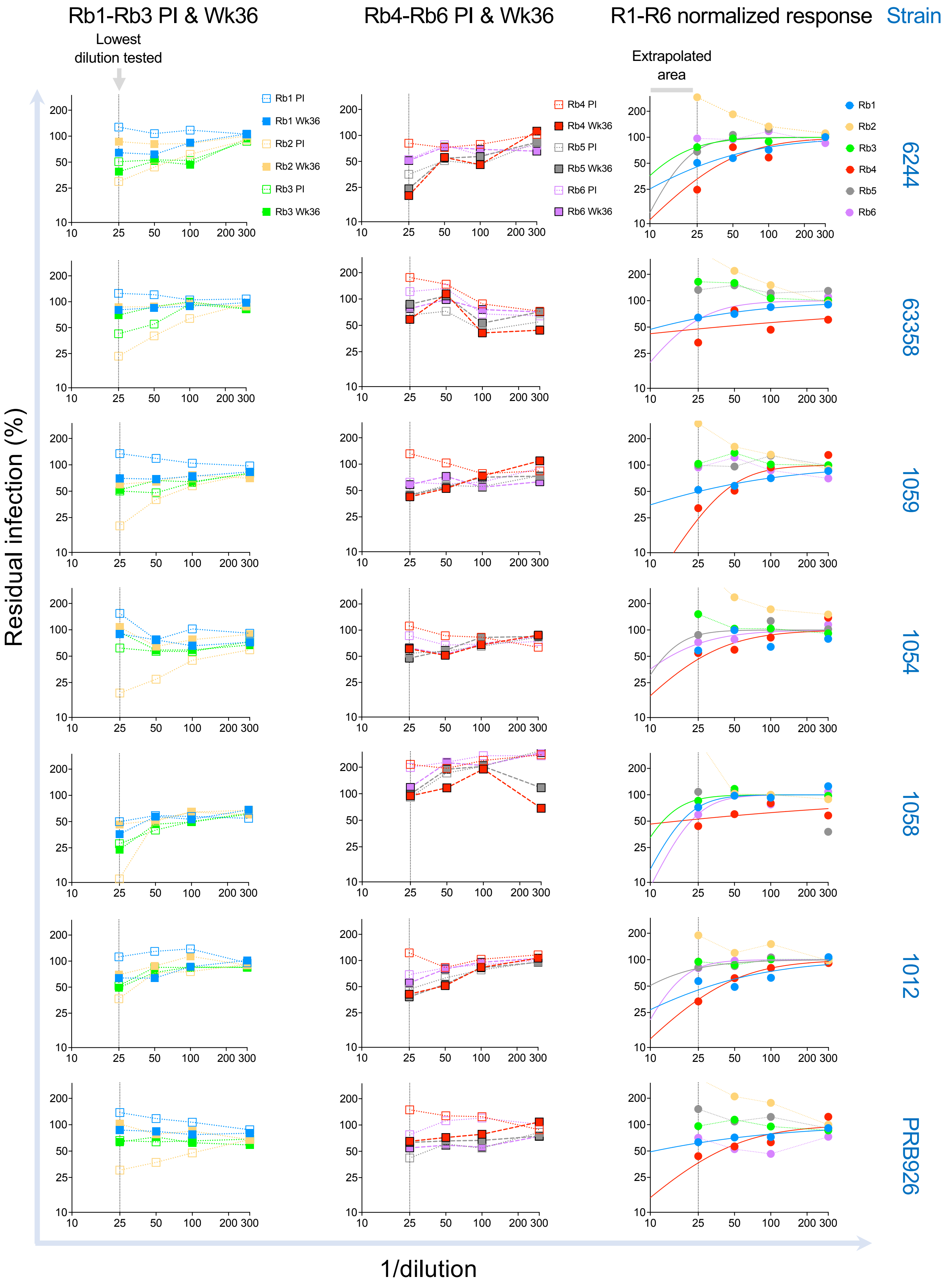

Supplementary Figure 7. Neutralization profile of pre-immune (PI) and Week 36 (Wk36) post immunization sera of rabbits 1-6 (legend on next page).

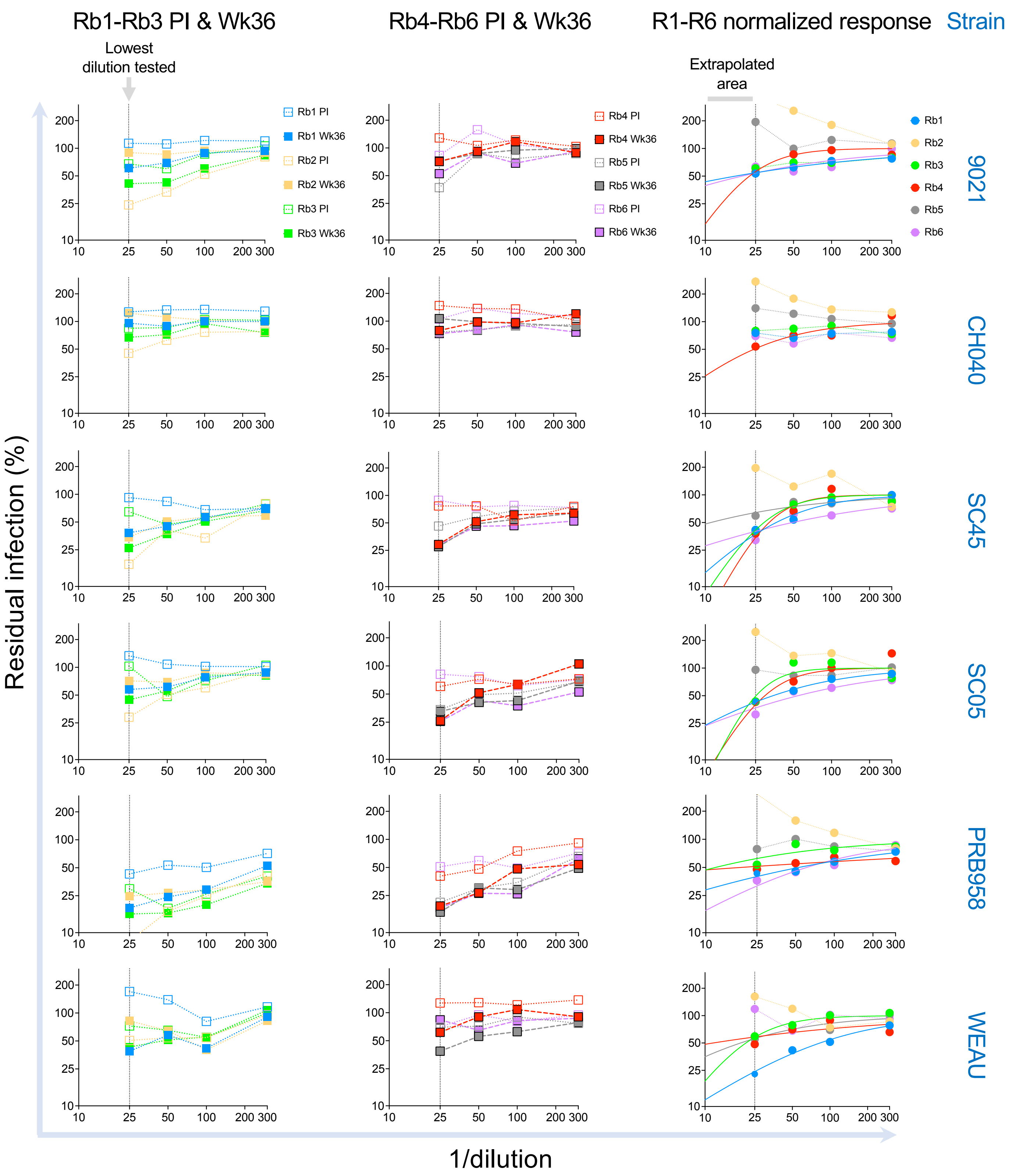

Supplementary Figure 7. Neutralization profile of pre-immune (PI) and Week 36 (Wk36) post immunization sera of rabbits 1-6. We measured the sensitivity of viruses pseudotyped with 13 transmitted / founder Envs to sera of 6 immunized rabbits by incubating specified pseudoviruses with sera collected 36 weeks post immunization and separately, as reference (control), with pre-immune (PI) sera. Each dilution of week 36 sera (1:25, 1:50, 1:100 and 1:300) has the matched PI control. We then added TZM-bl target cells and measured virus infectivity after 48 hours. Left and middle plots, effects of PI and week 36 sera of rabbits 1-6 on pseudovirus infectivity expressed as percent of no-sera infection. Right plots, response of week 36 sera of rabbits 1-6 normalized to the response of the related PI for each dilution tested [normalized response (X dilution) = residual infection (week 36; X dilution) / residual infection (PI; X dilution)*100]. Response was non-linearly fitted to the logistics equation and extrapolated area below 1:25 dilution (lowest tested; dashed line) is indicated. Normalized data that could not be fitted is shown with dashed lines.

a

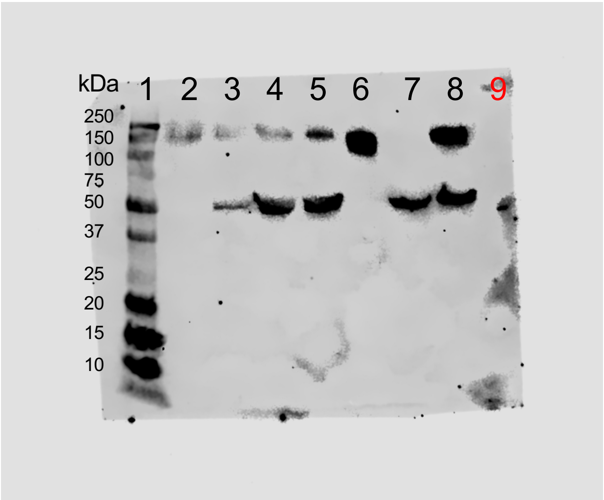

1 = molecular weight protein markers
2 = supernatant of cells transfected with mRNA HIV-1$_{AD8}$ Env + mRNA HIV-1 Gag 2:1 ratio
3 = supernatant of cells transfected with mRNA HIV-1$_{AD8}$ Env + mRNA HIV-1 Gag 1:1 ratio
4 = lysate of cells transfected with mRNA HIV-1$_{AD8}$ Env + mRNA HIV-1 Gag 2:1 ratio
5 = lysate of cells transfected with mRNA HIV-1$_{AD8}$ Env + mRNA HIV-1 Gag 1:1 ratio
6 = purified HIV-1$_{AD8}$ gp120
7 = purified HIV-1 Gag
8 = mixture of purified HIV-1$_{AD8}$ gp120 + HIV-1 Gag (this lane is not shown in figure 1d)
9 = empty
Controls = lanes 6, 7 & 8

b

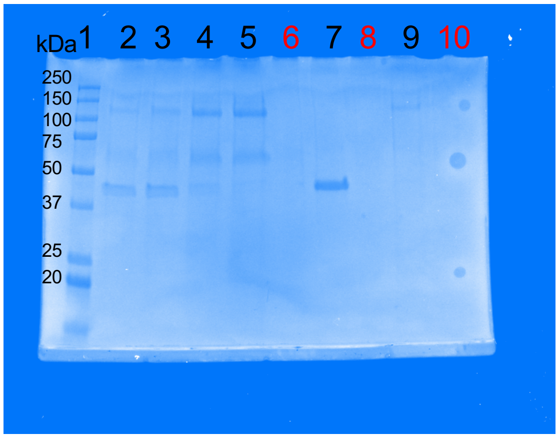

1 = molecular weight protein markers
2 = spycatcher VLP : 1059-Spytag 1:1 ratio
3 = spycatcher VLP : 1059-Spytag 1:2 ratio
4 = spycatcher VLP : 1059-Spytag 1:4 ratio
5 = spycatcher VLP : 1059-Spytag 1:6 ratio
6 = empty
7 = purified spycatcher VLP
8 = empty
9 = purified 1059-Spytag
10 = empty
Controls = lanes 7 & 9

c

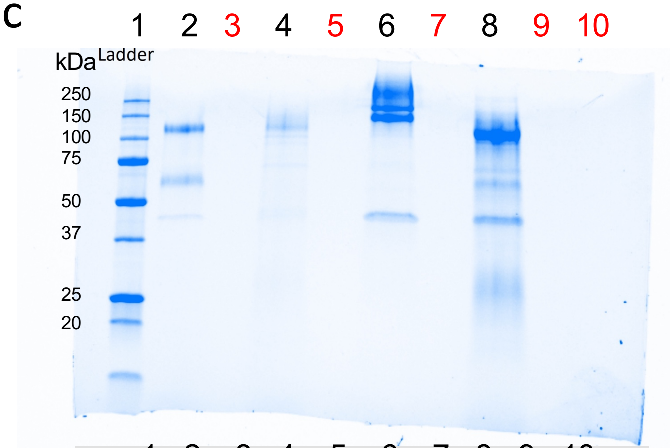

1 = molecular weight protein markers
2 = purified synVLP-1059
3 = empty
4 = purified 1059-Spytag
5 = empty
6 = unpurified synVLP-1059 (w/o reducing agent)
7 = empty
8 = unpurified synVLP-1059 (with reducing agent)
9 = empty
10 = empty
Controls = lanes 4, 6 & 8
Only lanes 1+2 are shown in Figure 1g

d

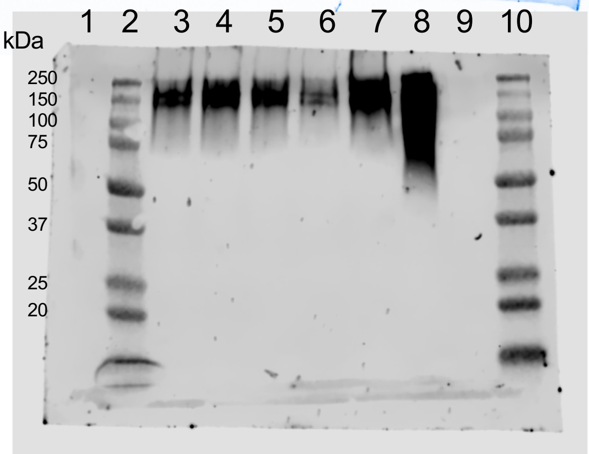

1 = empty
2 = molecular weight protein markers
3 = lysate of cells transfected with 4 µg mRNA HIV-1$_{AD8}$ Env transcribed using pseudouridine
4 = lysate of cells transfected with 6 µg mRNA HIV-1$_{AD8}$ Env
5 = similar to lane 4 but 4 µg mRNA
6 = similar to lane 4 but 1.6 µg mRNA
7 = lysate of cells transfected with 1.6 µg mRNA cytoplasmic tail-deleted HIV-1$_{AD8}$ Env
8 = purified HIV-1$_{AD8}$ gp120
9 = control: untransfeced 293T cells
10 = molecular weight protein markers
Controls = lanes 8 & 9

Supplementary Figure 8. Full and un-cropped SDS-PAGE gels / western blots used in Figures 1d (a), 1e (b), 1g (c) and Supplementary Fig. 5b (d). Lanes labeled with red numbers were kept empty. Precision Plus Protein Dual Color Standards (Bio-Rad; catalog number 1610374) was used as size standards.
